# Isorhynchophylline alleviates learning and memory impairments induced by aluminum chloride in mice

**DOI:** 10.1186/s13020-018-0187-8

**Published:** 2018-06-14

**Authors:** Hui-Qin Li, Siu-Po Ip, Guo-Qing Zheng, Yan-Fang Xian, Zhi-Xiu Lin

**Affiliations:** 1 School of Chinese Medicine, Faculty of Medicine, The Chinese University of Hong Kong, Hong Kong, SAR People’s Republic of China; 2 Brain Research Centre, School of Chinese Medicine, Faculty of Medicine, The Chinese University of Hong Kong, Hong Kong, SAR People’s Republic of China; 3 Department of Neurology, The Second Affiliated Hospital and Yuying Children’s Hospital of Wenzhou Medical University, Wenzhou, Zhejiang Province People’s Republic of China; 4 Institute of Integrative Medicine, Faculty of Medicine, The Chinese University of Hong Kong, Hong Kong, SAR People’s Republic of China

**Keywords:** Isorhynchophylline, Aluminum chloride, Learning and memory impairments, Oxidative stress, Cholinergic system, Nuclear factor kappa B pathway

## Abstract

**Background:**

To evaluate the effect of Isorhynchophylline (IRN) on the learning and memory impairments induced by aluminum chloride (AlCl_3_) in mice.

**Methods:**

Fifty male Balb-c mice (4-month-old) were randomly divided into five groups: control, AlCl_3_ plus vehicle, AlCl_3_ plus IRN (20 mg/kg), AlCl_3_ plus IRN (40 mg/kg) and AlCl_3_ plus donepezil (5 mg/kg). Learning and memory impairments were induced in mice by subcutaneously injecting with AlCl_3_ (50 mg/kg) once a day for 8 consecutive weeks. At the same time, mice were intragastrically given vehicle or IRN (20 and 40 mg/kg) or donepezil (5 mg/kg) 30 min before each AlCl_3_ injection. The spatial learning and memory function was assessed using radial arm maze. After sacrificed, the parameters of oxidative stress and cholinergic system in the brain tissues were examined with ELISA kits. Moreover, the expression of nuclear factor kappa B (NF-κB) signaling pathway was analyzed with western blotting.

**Results:**

The results showed that treatment with IRN could significantly ameliorate the cognitive deficits induced by AlCl_3_ in mice. In addition, treatment with IRN was found to reduce the level of malondialdehyde, enhance the activities of superoxide dismutases and catalase, increase the level of glutathione, and markedly inhibit the activity of acetylcholinesterase (AChE) in the brain tissues of the AlCl_3_-treated mice. Moreover, IRN significantly suppressed the phosphorylation of NF-κB p65 and IκBα in the brain tissues of AlCl_3_-treated mice. However, IRN did not show significant effect on the activity of butyrylcholinesterase.

**Conclusion:**

Our findings demonstrated for the first time that IRN could alleviate learning and memory impairments induced by AlCl_3_ in mice. The neuroprotective effect of IRN against AlCl_3_-induced AD is probably mediated, at least in part, through inhibiting the AChE activity and reducing the oxidative damage of brain tissue via suppress the NF-κB signaling pathway. These results contributed to a better understanding of the in vivo anti-AD mechanism of IRN. It was concluded that IRN could protect the learning and memory function.

**Electronic supplementary material:**

The online version of this article (10.1186/s13020-018-0187-8) contains supplementary material, which is available to authorized users.

## Background

Alzheimer’s disease (AD) is a neurodegenerative disease characterized by progressive memory dysfunction and cognitive deficiency [1]. As the world’s population ages, we face a looming global epidemic of AD; and by 2050 the number of AD patients is estimated to be 106.4 million globally [2]. Although beta-amyloid (Aβ) accumulation and tau protein malformation are generally accepted to be the causal factors of AD, the cellular and molecular pathology and etiology of AD have not been fully understood, largely due to the fact that AD is a multifactorial disease involving genetic, epigenetic, and environmental factors [3]. Furthermore, present treatments available for AD are usually limited to symptomatic management, and no satisfactory therapeutic agents are available for AD patients [4–6]. Thus, novel therapies with better efficacy and safety profiles are urgently needed to modify the natural history of AD.

Aluminum, which can cross the blood brain barrier (BBB) [7], is a well-known neurotoxin that has close association with AD pathogenesis [8]. Based on the studies on the hippocampal neurons of AD patients, aluminum was found to be involved in the formation of neurofibrillary tangles in neurons, and therefore considered as a causative factor for AD [9]. Moreover, studies have also shown that exposure to aluminum can cause memory impairment in animal models [10, 11]. The underlying mechanisms for aluminum to induce AD-like behaviors are believed to involve oxidative damage [11], formation of hyperphosphorylated tau [12], aggregation of amyloid beta protein [13], increase in AChE activity [14] and neuronal apoptosis [13]. In addition to neuronal dysfunction, aluminum could also lead to brain dysfunction via glial alterations [15]. Given that aluminum exposure could eventually result in neuropathological and neurobehavioral changes, and impaired learning and memory, AlCl_3_-induced model is therefore used as an established AD animal model and often used for testing the efficacy of therapeutic agents for AD.

**Fig. 1:**
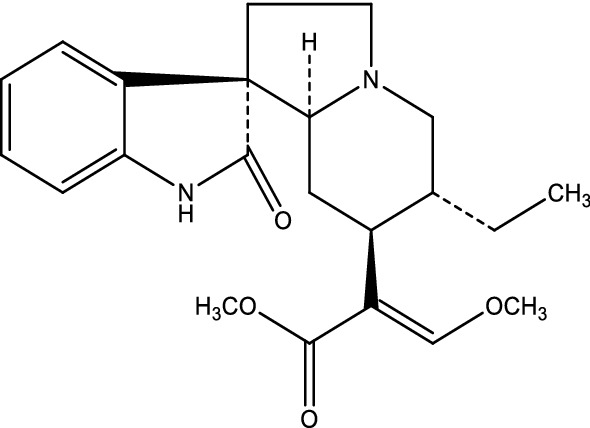
Chemical structure of isorhynchophylline (IRN)

*Uncaria rhynchophylla* is a component herb of many popular herbal formulae commonly prescribed for the treatment of AD [16, 17]. Recently, many studies have showed that ethanol extract of *U. rhynchophylla* has neuroprotective effect on different in vivo and in vitro AD models [18, 19]. Isorhynchophylline (IRN, the chemical structure is shown in Fig. 1), a c-22 oxindole alkaloid richly presented in the ethanol extract of *U. rhynchophylla*, is the main active chemical ingredient for its biological activities [20]. Our previous studies revealed that IRN could ameliorate the neurotoxicity induced by Aβ_25–35_ on PC12 cells via PI3K/Akt/GSK-3β signaling pathway [21], improve the memory deficits induced by Aβ_25–35_ in rats via inhibition of neuronal apoptosis and tau protein hyperphosphorylation [22], and reduce the memory deficits induced by d-galactose in mice through enhancing the antioxidant status and anti-inflammatory effect in brain tissues via nuclear factor kappa B (NF-κB) signaling pathway [23]. Moreover, it was found that the LD_50_ values of IRN in mice are about 510, 217 and 80 mg/kg, respectively, with oral, intraperitoneal and intravenous administration, indicating that IRN is at least 10 times less toxic than other known anti-AD drugs, and is a safer chemical compound for AD treatment [24]. All these studies implied that IRN is a promising neuroprotective agent for AD treatment warranting further investigation. However, whether IRN has therapeutic effect on AlCl_3_-treated mice and the underlying mechanisms have not been studied yet. The present study aimed to investigate the neuroprotective effects of IRN on cognitive function in the AlCl_3_-treated mice and to elucidate the underlying mechanisms of action.

## Methods

The minimum standards of reporting checklist (Additional file 1) contains details of the experimental design, statistics, and resources used in this study.

### Drugs and reagents

Isorhynchophylline (IRN, purity ≥ 98%) was purchased from Chengdu Mansite Pharmaceutical Co. Ltd. (Chengdu, Sichuan, China). Aluminum chloride hexahydrate (AlCl_3_) and donepezil hydrochloride (referred to simply as donepezil thereafter) were purchased from Sigma-Aldrich (St. Louis, MO, USA). All other reagents and chemicals used in the study were of analytical grade.

### Animals

Fifty male Balb-c mice (4-month-old, weighing 25–30 g) were obtained from the Laboratory Animal Services Center, The Chinese University of Hong Kong. The animals were maintained on a 12 h light/dark cycle under controlled temperature (22 ± 2 °C) and humidity (50 ± 10%), and given standard diet and water ad libitum.

### Animal groupings and drug treatment

**Fig. 2:**
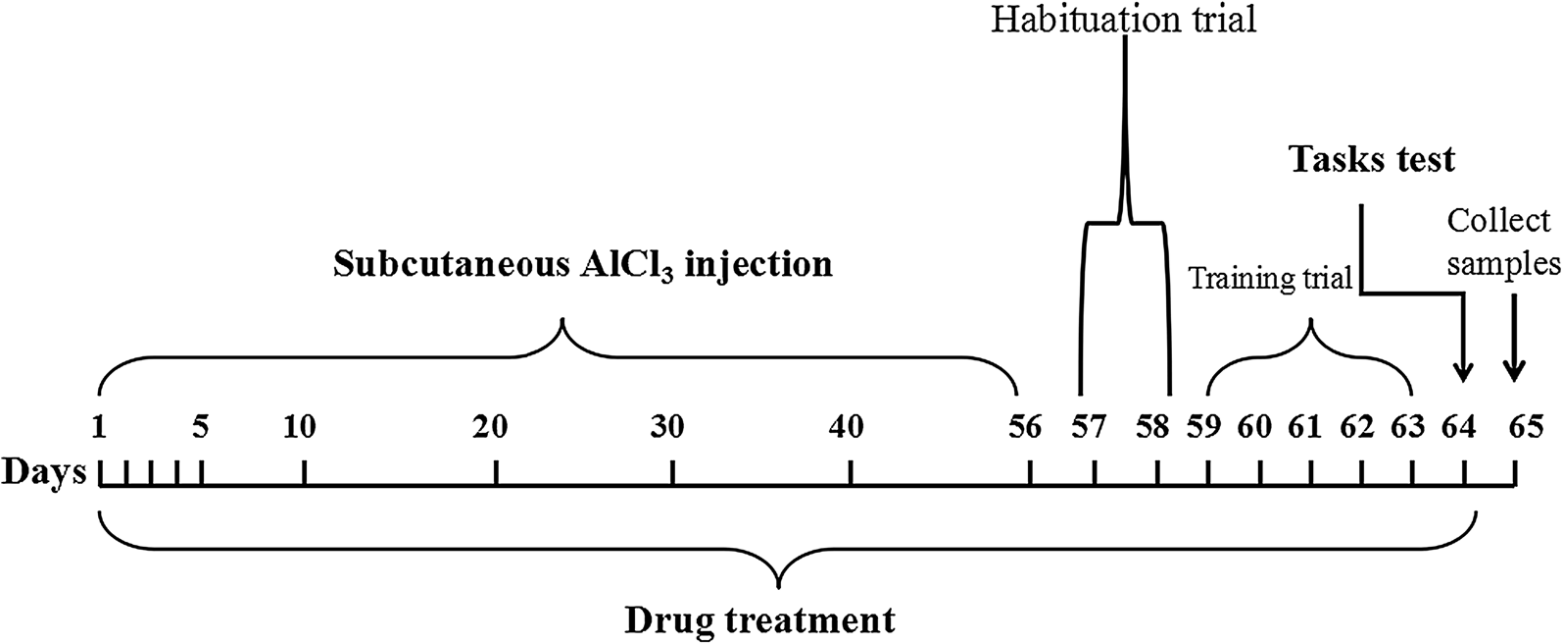
Experimental design and treatment schedule

The mice were randomly assigned into five groups of 10 animals each: (a) control; (b) AlCl_3_ plus vehicle; (c) and (d) AlCl_3_ plus IRN (20 and 40 mg/kg); and (e) AlCl_3_ plus donepezil (5 mg/kg), which was used as the positive control [22]. The dosages of IRN were selected based on our previous studies [22, 23]. AlCl_3_ was dissolved in sterile physiological saline at the concentration of 5 mg/mL, and subcutaneously injected into the mice at a dosage of 50 mg/kg once a day for 8 consecutive weeks before the behavioral assessments were started. The duration and dosage of aluminum was selected based on the previous published studies with minor modification [25]. In the control group, mice were subjected to the same injection schedule except using physiological saline instead of AlCl_3_. At the same time, IRN and donepezil were suspended in 0.5% sodium carboxymethyl cellulose (CMC-Na), then given to the mice intragastrically daily 30 min before each AlCl_3_ injection, while the control group and AlCl_3_ plus vehicle control group were given the same volume of 0.5% CMC-Na. Behavioral assessments were carried out after the AlCl_3_ injection was finished. During the behavioral assessment, the treatment was ongoing until the working and reference memory tasks test, which was undertaken at day 64. Detailed experimental schedules were depicted in Fig. 2.

### Radial arm maze (RAM) test

Spatial learning and memory changes in mice were determined by the RAM test. A modular radial arm maze with SuperMaze V2.0 video tracking software (Shanghai Xinruan Information Technology Co. Ltd, Shanghai, China) was used for this experiment. The maze consisted of eight arms, numbered from 1 to 8 (35 cm long, 5 cm wide and 10 cm high), extending radially from a central platform (22 cm in diameter). The radial arm maze test was conducted as described previously with minor modifications [26, 27]. In the room, several visual extra-maze cues were provided close (10–30 cm) to the maze in a fixed configuration. A circular food well (1 cm in diameter, 0.5 cm deep) was positioned at the end of each arm. During behavioral training and testing, to stimulate hunger, the animals were kept on restricted diet with only water being available ad libitum and the body weight was maintained at about 85–90% of their free-feeding level. Initially, animals received two 10 min habituation trials, 24 h apart, with free access to all arms on the first 2 days. At the beginning of each habituation trial, three or four mice were simultaneously placed in the central platform and all arms were baited with several approximately 10 mg food pellets. Then, mice were trained for 5 consecutive days, one trial per day, to run to the end of the arms and consume the bait. At the beginning of each training trial, only one mouse was placed in the central platform and only four constant arms were randomly selected and baited with one approximately 10 mg food pellet, which was placed in a food cup that prevented visual detection. A trial was ended when all the four baits had been consumed or after a maximum of 10 min had elapsed. An arm entry was counted when all four limbs of the mice were within an arm. At the eighth day, the mice were subjected to working and reference memory tasks, in which the same four arms were baited as each daily training trial, and the following behavioural measurements were recorded: (1) number of reference memory errors (RMEs), i.e. entries into a non-baited arm, (2) number of working memory errors (WMEs), i.e. re-entries into an already visited baited arm, (3) number of total entries to complete the test.

### Preparation of brain samples

Twenty-four hours after the RAM test, mice were decapitated under anesthesia and the brain tissues were removed quickly. After washed with normal saline over the ice, brains were immediately stored at − 80 °C until assay.

### Measurement of oxidative stress

To evaluate the effect of IRN on oxidative stress status, the levels of malondialdehyde (MDA) and glutathione (GSH) and the activities of superoxide dismutases (SOD) and catalase (CAT) were measured. For these biochemical analyses, 10% (w/v) brain homogenate was prepared in 0.9% sterile normal saline using a potter homogenizer at a speed of 1200 rpm. The homogenate was centrifuged at 3000×*g* for 10 min at 4 °C and then the supernatant was collected. The supernatant was separated and tested with SOD assay kit, GSH assay kit, CAT assay kit and MDA assay kit (Cat. A001-3, A006-1, A007-1 and A003-1, respectively, Nanjing Jiancheng Institute, Jiangsu, China) following the manufacturer’s protocols. CAT activity was determined by the rate of decomposition of 1 μM H_2_O_2_ and expressed as U/mgprot. SOD activity was determined by the amount of enzyme required to produce 50% inhibition and expressed as U/mgprot. Reduced GSH was determined based on the formation of a yellow colored complex with 5,5′-dithiobis-(2-nitrobenzoic acid) (DTNB) and the level of GSH in tissue was expressed as mgGSH/gprot. Reduced MDA was determined based on the formation of a red colored complex with thiobarbituric acid (TBA) and the level of MDA in tissue was expressed as nmol/mgprot. All samples were measured in duplicate.

### Measurement of the activities of acetylcholinesterase (AChE) and butyrylcholinesterase (BuChE)

The activities of AChE and BuChE in the brain tissues were examined. At first, 10% (w/v) brain homogenate was prepared in 0.9% sterile normal saline using a potter homogenizer at a speed of 1200 rpm. The homogenate was centrifuged at 3000×*g* for 10 min at 4 °C and then the supernatant was collected. The supernatant was separated and tested with the AChE assay kit and BuChE assay kit (Cat. A024 and A025, respectively, Nanjing Jiancheng Institute, Jiangsu, China) following the manufacturer’s instructions. The activities of AChE and BuChE were determined based on the formation of Sym-Trinitrobenzene (TNB), which is a yellow colored complex, and the results were both expressed as U/mg protein. All samples were measured in duplicate.

### Western blotting analysis

For preparation of whole cell protein lysate, the brain tissues were homogenized in RIPA lysis buffer (50 mM Tris–HCl (pH 8.0), 150 mMNaCl, 1% NP-40, 0.1% SDS, 0.5% sodium deoxycholate and 1% Protease/Phosphatase Inhibitor Cocktail) (Cell Signaling Technology, USA) for 30 min on ice, then centrifuged at 12,000 rpm at 4 °C, and then the supernatant was collected. Protein concentrations were determined using Bradford method with protein assay dye reagent (Bio-Rad, USA). Equal amounts of protein of different samples were separated by sodium dodecyl sulfate polyacrylamide gel electrophoresis (SDS-PAGE), and then transferred to PVD membranes. After blocked with 5% (w/v) non-fat milk in TBS-T (50 mM Tris, 150 mM NaCl and 0.1% Tween-20, pH 7.4) at room temperature for 2 h, the PVD membranes were incubated overnight at 4 °C in NF-κB p65 (p65), phospho-NF-κB p65 (p-p65), phospho-IκBα (p-IκBα), IκBα or β-actin primary antibody (1:1000, Cell Signaling Technology, #6956S, 3033S, 2859S, 9242S or 3700S, respectively). Rinsed with TBS-T for 5 min × 3 times, the PVD membranes were then incubated with secondary antibody for 2 h at room temperature, and then rinsed again with TBS-T for 5 min × 3 times. The protein bands were visualized by the ECL western blotting detection reagents (Amersham Biosciences, Buckinghamshire, UK). The intensity of each band was analyzed using Image J software (NIH Image, MD, USA).

### Statistical analysis

All data were presented as the mean ± standard error of the mean (SEM). Multiple group comparisons were performed using one-way analysis of variance (ANOVA) followed by Post-hoc Dunnett’s test to detect inter-group differences. GraphPad Prism software (Version 5, GraphPad Software, Inc., CA, USA) was used to perform the statistical analysis. A difference was considered statistically significant when the *p *< 0.05.

## Results

### IRN ameliorated the cognitive deficits induced by AlCl_3_ in mice

**Fig. 3:**
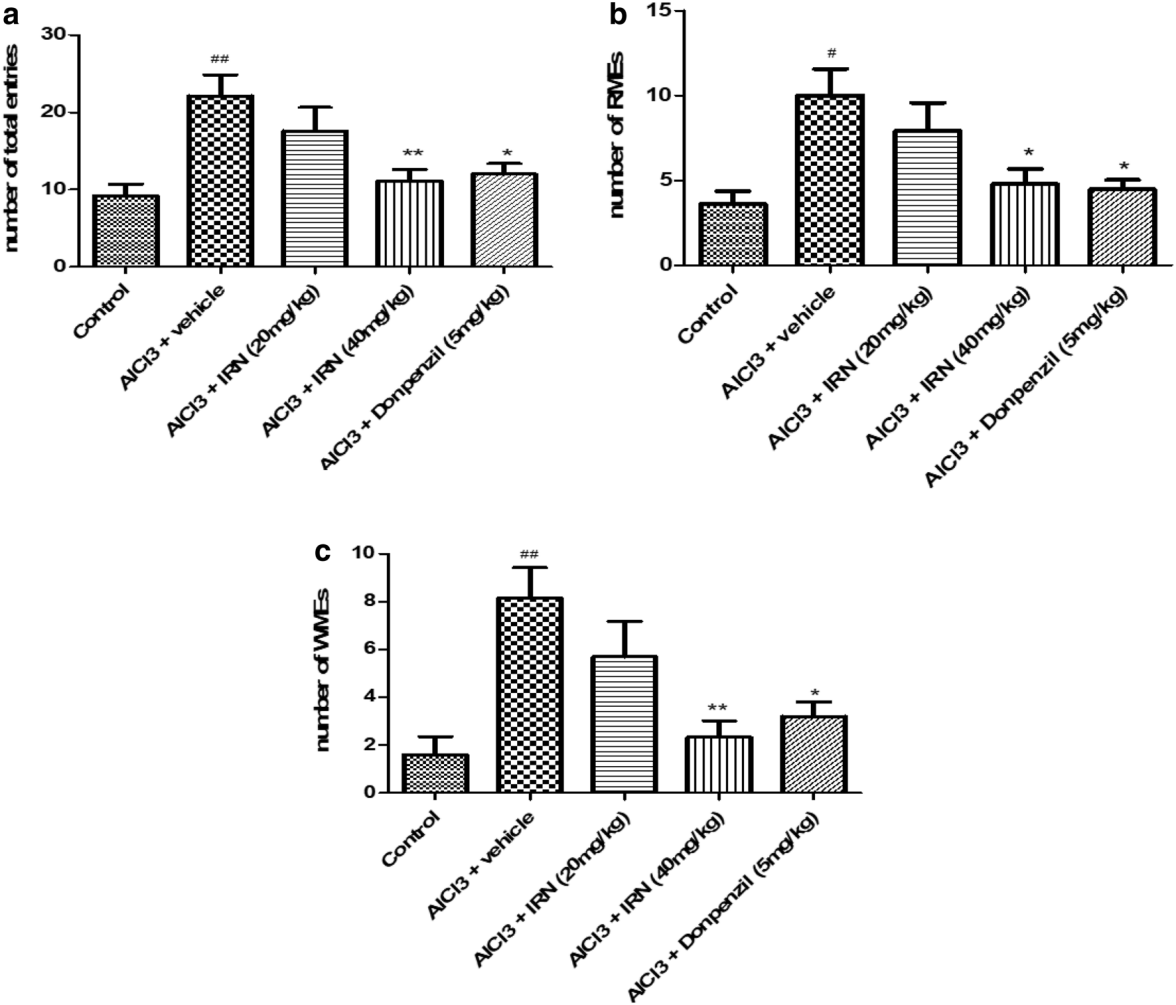
Effects of IRN on spatial learning and memory of mice as evaluated by the RAM test after 7 days of training. The total entrances to complete the task (**a**), the reference memory errors (RMEs) (**b**), and the working memory errors (WMEs) (**c**). Data were expressed as mean ± SEMs (n = 6–10). ^#^*p *< 0.05 and ^##^*p *< 0.01 compared with the control group; *p < 0.05 and **p < 0.01 compared with the AlCl_3_ + vehicle group

After 7 days of training, the mice were subjected to working and reference memory tasks. Figure 3a shows the effect of IRN on the number of total entries to complete the maze. The results showed that AlCl_3_ + vehicle group markedly increased the number of total entries when compared with the control group (F (4, 33) = 4.694, *p* < 0.01), while treatment with IRN (40 mg/kg) significantly decreased the number of total entries when compared with the AlCl_3_ + vehicle group (*p* < 0.01). Donepezil (5 mg/kg) treatment also decreased the number of total entries (*p* < 0.05) when compared with the AlCl_3_ + vehicle group.

The effects of IRN on reference memory errors (RMEs) and the working memory errors (WMEs) were shown in Fig. 3b, c, respectively. The results showed that AlCl_3_ plus vehicle group elevated the RMEs (F (4, 33) = 3.895, *p *< 0.05) and WMEs (F (4, 33) = 4.416, *p *< 0.01) to complete the task when compared with the control group, while treatment with IRN (40 mg/kg) significantly reduced the RMEs (*p *< 0.05) and WMEs (*p *< 0.01) when compared with the AlCl_3_ + vehicle group. Donepezil (5 mg/kg) treatment also significantly reduced the RMEs (*p *< 0.05) and WMEs (*p *< 0.05) when compared with the AlCl_3_ + vehicle group. On the other hand, IRN treatment at dosage of 20 mg/kg/day showed no significant difference in the number of the total entries (*p *> 0.05), RMEs (*p *> 0.05) or WMEs (*p *> 0.05) when compared with the AlCl_3_ + vehicle group.

### IRN reduced the oxidative damage induced by AlCl_3_ in mice

**Fig. 4:**
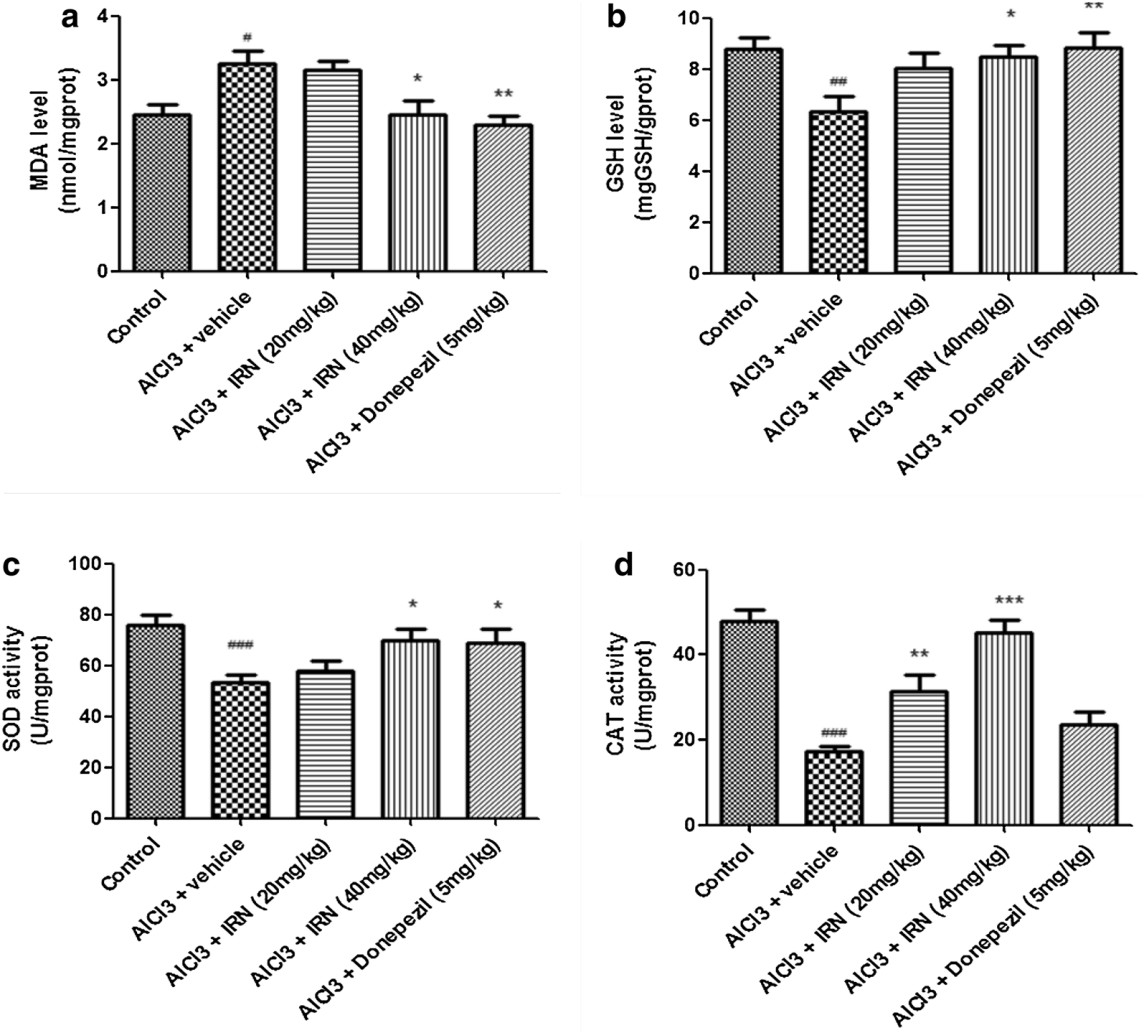
Effect of IRN treatment on the levels of MDA (**a**) and GSH (**b**) and the activities of SOD (**c**) and CAT (**d**) in the brains of AlCl_3_-treated mice. Data were expressed as mean ± SEMs (n = 6–9). ^#^*p *< 0.05, ^##^*p *< 0.01 and ^###^*p *< 0.001 compared with the control group; *p < 0.05, **p < 0.01 and ***p < 0.001 compared with the AlCl_3_ + vehicle group

The oxidative stress of brain tissue was assessed by measuring the level of MDA, a lipid peroxidation product (Fig. 4a), and antioxidants including GSH (Fig. 4b), SOD (Fig. 4c) and CAT (Fig. 4d).

Figure 4a showed that the MDA level was significantly higher in AlCl_3_ + vehicle group than the control group (F (4, 34) = 6.585, *p *< 0.05], while treatment with IRN (40 mg/kg) significantly decreased the MDA level when compared with the AlCl_3_ + vehicle group (*p *< 0.05). Donepezil (5 mg/kg) treatment also inhibited the MDA level when compared with AlCl_3_ + vehicle group (*p *< 0.01).

As shown in Fig. 4b, the GSH level was significantly lower in the AlCl_3_ + vehicle group than the control group (F (4, 34) = 4.06, *p *< 0.01), while treatment with IRN (40 mg/kg) significantly increased the GSH level when compared with the AlCl_3_ + vehicle group (*p *< 0.05). Donepezil (5 mg/kg) treatment also increased the GSH level when compared with the AlCl_3_ + vehicle group (*p *< 0.01).

Figure 4c showed that the SOD activity was significantly suppressed in the AlCl_3_ + vehicle group when compared with the control group (F (4, 34) = 5.571, *p *< 0.001), while treatment with IRN (40 mg/kg) significantly increased the SOD activity when compared with the AlCl_3_ + vehicle group (*p *< 0.05). Donepezil (5 mg/kg) treatment also increased the SOD activity when compared with the AlCl_3_ + vehicle group (*p *< 0.05).

Moreover, Fig. 4d showed that the CAT activity was significantly decreased in the AlCl_3_ + vehicle group when compared with the control group (F (4, 34) = 18.29, *p *< 0.001), while the treatment with IRN (20 and 40 mg/kg) significantly increased the CAT activity when compared with the AlCl_3_ + vehicle group, (*p *< 0.01 and *p *< 0.001, respectively). However, donepezil (5 mg/kg) treatment did not show significant effect (*p *> 0.05) on the activity of CAT.

### IRN inhibited the cholinesterase activity in the brain of AlCl_3_-treated mice

**Fig. 5:**
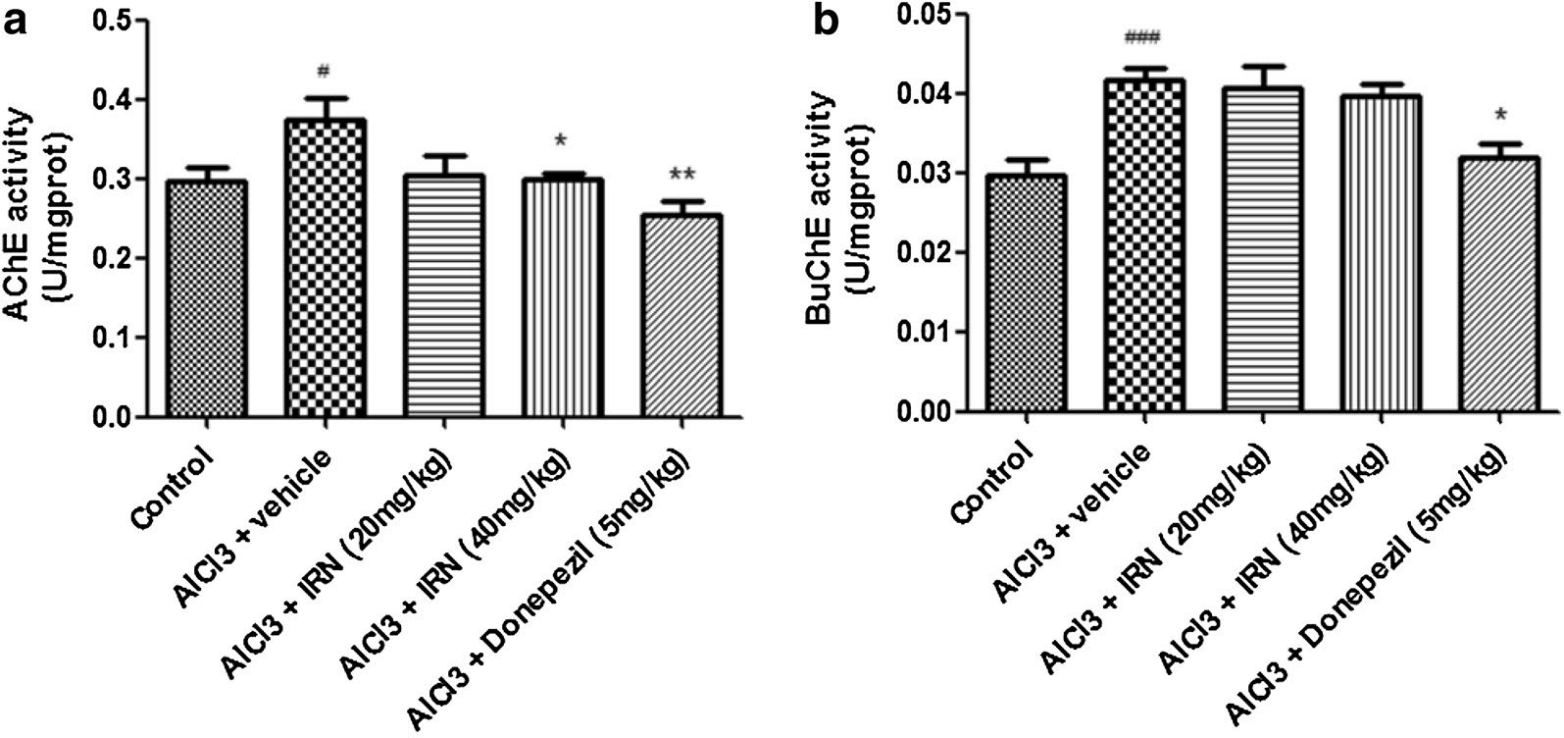
Effect of IRN treatment on the activities of AChE (**a**) and BuChE (**b**) in the brain of the AlCl_3_-treated mice. Data were expressed as mean ± SEMs (n = 6–9). ^#^*p *< 0.05 and ^###^*p *< 0.001 compared with the control group; *p < 0.05 and **p < 0.01 compared with the AlCl_3_ + vehicle group

The effect of IRN on cholinesterase activity was assessed by measuring the activities of AChE (Fig. 5a) and BuChE (Fig. 5b). Figure 5a showed that the AChE activity was significantly increased in the AlCl_3_ + vehicle group when compared with the control group (F (4, 34) = 4.144, *p *< 0.05], while treatment with IRN (40 mg/kg) significantly decreased the AChE activity when compared with the AlCl_3_ + vehicle group (*p *< 0.05). Donepezil (5 mg/kg) treatment also decreased the AChE activity when compared with the AlCl_3_ + vehicle group (*p *< 0.01). On the other hand, Fig. 5b showed that the BuChE activity was significantly increased in the AlCl_3_ + vehicle group when compared with the control group, but treatment with IRN (20 and 40 mg/kg) did not show significant effect on BuChE activity when compared with the AlCl_3_ + vehicle group (*p *> 0.05 for both). Donepezil (5 mg/kg) treatment markedly decreased the BuChE activity when compared with the AlCl_3_ + vehicle group (*p *< 0.05).

### IRN inhibited the activation of NF-κB signaling pathway in the brain of AlCl_3_-treated mice

**Fig. 6:**
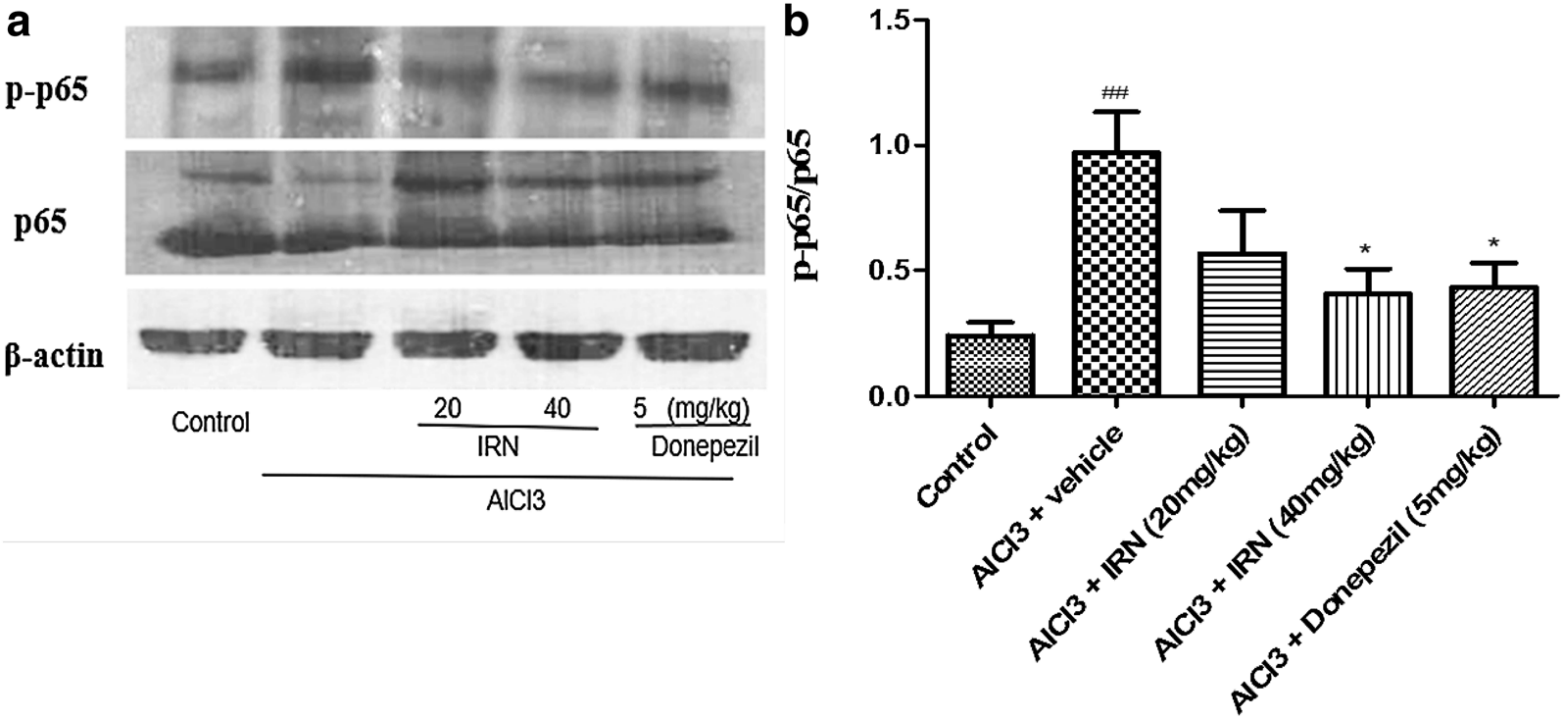
Effect of IRN treatment on NF-κB p65 (p65) and phospho-NF-κB p65 (p-p65) expressions in the brain of the AlCl_3_-treated mice were determined by Western blotting (**a**). The relative density of p-p65/p65 (**b**) were analyzed by Image J. software. Data were expressed as mean ± SEMs (n = 4–6). ^###^*p *< 0.01 compared with the control group; **p < 0.05 compared with the AlCl_3_ + vehicle group

**Fig. 7:**
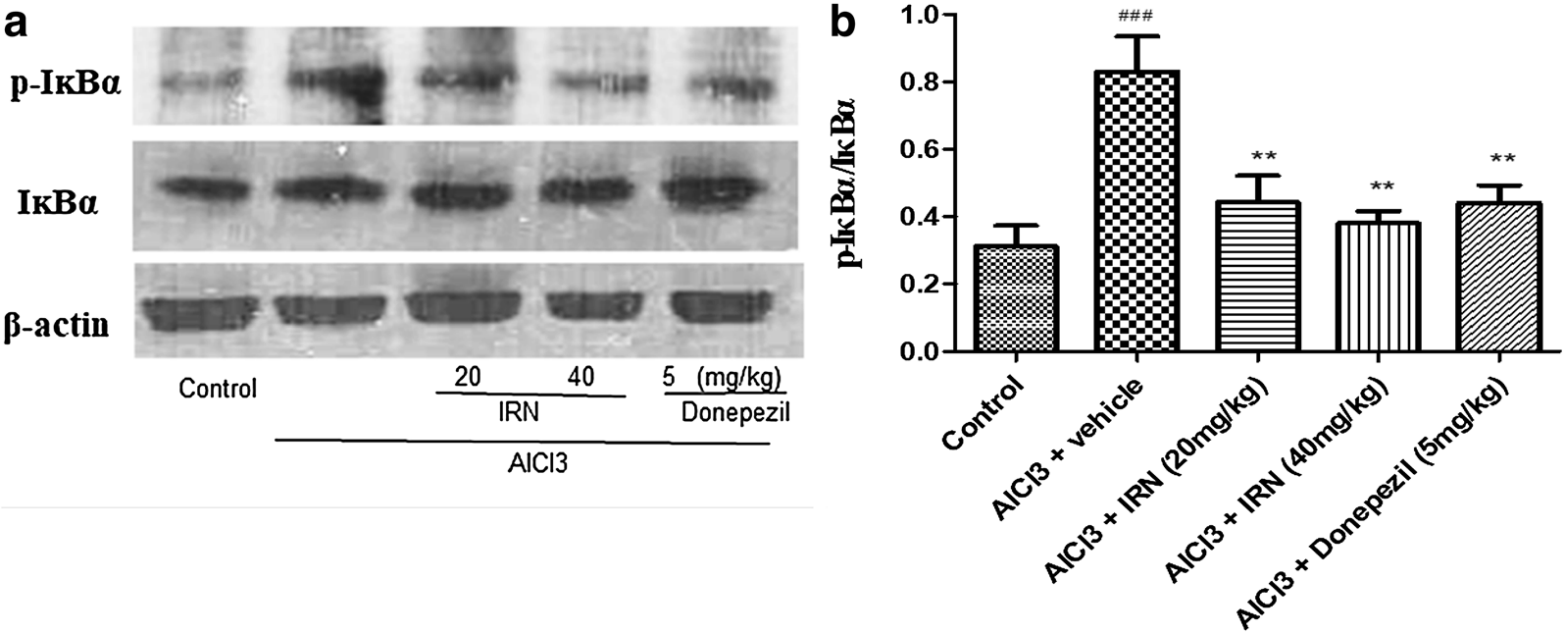
Effect of IRN treatment on IκBα and phospho-IκBα (p-IκBα) expressions in the brain of the AlCl_3_-treated mice were determined by Western blotting (**a**). The relative density of p-IκBα/IκBα (**b**) were analyzed by Image J. software. Data were expressed as mean ± SEMs (n = 4–6). ^###^*p *< 0.001 compared with the control group; **p < 0.01 compared with the AlCl_3_ + vehicle group

Effect of IRN treatment on the NF-κB signaling pathway in the brain tissues of AlCl_3_-treated mice were determined by Western blotting (Figs. 6a and 7a). Relative density of p-p65/p65 and p-IκBα/IκBα were shown in Figs. 6b and 7b, respectively. Figure 6b showed that the relative density of p-p65/p65 was significantly increased in the AlCl_3_ + vehicle group when compared with the control group (F (4, 34) = 9.896, *p *< 0.001], while treatment with IRN (20 and 40 mg/kg) significantly decreased the relative density of p-p65/p65 when compared with the AlCl_3_ + vehicle group (*p *< 0.01 and *p *< 0.01, respectively). Donepezil (5 mg/kg) treatment also decreased the relative density of p-p65/p65 when compared with AlCl_3_ + vehicle group (*p *< 0.01). On the other hand, Fig. 7b showed that the relative density of p-IκBα/IκBα was significantly increased in the AlCl_3_ + vehicle group when compared with the control group (F (4, 23) = 7.125, *p *< 0.001), while the treatment with IRN (20 and 40 mg/kg) significantly decreased the relative density of p-p65/p65 when compared with the AlCl_3_ + vehicle group (*p *< 0.01 and *p *< 0.01, respectively). Donepezil (5 mg/kg) treatment also decreased the relative density of p-p65/p65 when compared with the AlCl_3_ + vehicle group (*p *< 0.01).

## Discussion

The present study investigated the neuroprotective effects of IRN on the AlCl_3_-induced cognitive impairment in mice. In our experiment, radial arm maze test was conducted to assess the spatial learning and memory functions of the mice. The results showed that AlCl_3_ treatment impaired the spatial learning and memory functions of the mice, enhanced the oxidative stress, damaged the cholinergic system, and activated the NF-κB signaling pathway in the brain. On the other hand, treatment with IRN significantly reversed these changes induced by AlCl_3_ in mice, suggesting that IRN is a potential anti-AD agent for the treatment of AD.

So far, several cognitive domains found disrupted in AD have been modeled, and the two most extensively modeled and assessed domains are working memory and reference memory [28]. The RAM test was firstly introduced by Olton in 1976 [29]. It is one of the most commonly used animal behavioral tasks to assess working memory and reference memory, just as the Morris water maze [28]. In the RAM test, the number of total entries to complete the maze is considered as an overall measure of learning and memory [30]. As previously reported, a reference memory error was noted if an animal enters an arm that never contained a food reward, and a working memory error was noted if an animal enters a baited arm previously visited during the same trial [31, 32]. The higher numbers of RMEs and WMEs are recorded, the worse the reference memory and working memory are indicated. In the present study, AlCl_3_ + vehicle group showed significant increases in RMEs and WMEs to complete the task when compared with the control group, indicating that AlCl_3_ induced significant impairments on reference memory and working memory in mice. IRN treatment reduced both of RMEs and WMEs when compared with the AlCl_3_ + vehicle group, indicating that IRN could improve the reference memory and working memory.

It has been reported that chronic exposure to aluminum at a dose of 1.6 mg/kg/day, equivalent to the high end of the human dietary aluminum range, could lead to oxidative damage in the brain and cause AD-like behaviors in rats [12]. However, short exposure to aluminum could only lead to transient memory impairment in Morris water maze [33], and low doses of aluminum may fail to observe impairment of performance in RAM test [34]. Therefore, to ensure well-established biochemical and behavioral deficits, suitable duration and dose of AlCl_3_ should be selected. In our preliminary study, we found that about 70% mice died after treatment with AlCl_3_ at a dose of 100 mg/kg/day for 8 consecutive weeks (data not shown), suggesting that the dose of AlCl_3_ (100 mg/kg/day) is too high. Therefore, in this present study, the dosage of 50 mg/kg/day of AlCl_3_ was used to induce the AD-like behaviors and the duration of exposure was set for 8 weeks.

Oxidative stress is an imbalance state between the generation and detoxification of reactive oxygen species (ROS) products [35]. It could be assessed by measuring peroxidation products of ROS, such as lipid peroxidation, and antioxidant levels [35]. Oxidative stress in human increases with age [36]. Oxidative stress is known to play an important role in the initiation of AD [37]. High lipid peroxidation and decreased antioxidant defenses are found to be presented in mild cognitive impairment (MCI) patients, implying that oxidative stress represents a sign of AD pathology and could be an early event in the progression of MCI to AD [38]. In addition, oxidative damage is found not only in the vulnerable neurons of brain affected in this disease [39], but also throughout the body in AD patients [40]. Moreover, many line of evidence showed that oxidative stress could cause marked accumulation of Aβ and phosphorylated tau protein in vitro and in vivo [41–43], which are considered to be the two main causal factors for AD. Given its close correlation, oxidative stress is even considered as a primary progenitor of AD, not merely an epiphenomenon. Thus, novel drug with anti-oxidative effects could be a preventative cure for the disease [44]. The present study showed that in the brains of the AlCl_3_ + vehicle mice, the MDA level was significantly higher, while the activities of SOD and CAT and the GSH level were significantly lower than control group, suggesting that AlCl_3_ was able to induce high oxidative stress in mice. However, IRN treatment decreased this oxidative damage induced by AlCl_3_, indicating it is a potential antioxidant for AD. In the present study, donepezil did not show effect on CAT activity. Donepezil is known as cholinesterase inhibitor for AD treatment. Although there is evidence that donepezil also affected the levels of GSH and MDA [45], but no evidence showed donepezil had effect on the activity of CAT, which is an antioxidant. Donepezil may suppress oxidative stress by affecting the levels of GSH and MDA, as well as the activity of SOD, but not CAT activity.

As is known, the cholinergic pathway in basal forebrain plays an important role in mnemonic processes, memory, conscious awareness and attention [46]. Evidence showed acetylcholine involved in spatial information processing [47]. Degeneration and loss of cholinergic neurons in the basal forebrain nuclei cause deficit of acetylcholine, then cause disturbances in presynaptic cholinergic terminals in the hippocampus and neocortex, leading to memory disturbances and other cognitive symptoms associated with AD [48]. Thus, acetylcholine is considered as an important neurotransmitter for memory in brains, and preventing the decrease of the level of acetylcholine may be an effective treatment strategy for AD. AChE is the primary cholinesterase that breaks down acetylcholine in the body, and activation of AChE will aggravate the deficit of acetylcholine. Cholinesterase inhibitors, such as donepezil, delay the breakdown of acetylcholine via suppressing the activity of AChE, more or less restore the cortical concentration of acetylcholine, and thus temporarily ameliorate the cognitive symptoms of AD [49]. Aluminum exposure was reported to be associated with impairment in the cholinergic system as a strong activator of AChE [50]. Our results showed that activity of AChE in the brain of AlCl_3_ + vehicle group was significantly higher than control group. On the other hand, IRN treatment significantly inhibited the AChE activity, but not BuChE. Although both of AChE and BuChE are cholinesterase, they differ in location, substrate specificity and kinetics [51]. IRN showed effect on AChE activity but not BuChE activity, indicating it is a potential selective AChE inhibitor for treatment of AD.

NF-κB, a collective name for inducible dimeric transcription factors, is involved in activation of many genes in response to infections, inflammation and other stressful situations requiring rapid reprogramming of gene expression [52]. Evidence showed that NF-κB is involved in brain function, particularly following injury and in neurodegenerative conditions such as AD, and is activated in neurons in certain regions of the brain during neurogenesis [53]. Furthermore, studies suggested that NF-κB plays a critical role in initiating and regulating the inflammation or oxidative stress in AD [54]. Increase of NF-κB p65 level could be found both in the temporal and frontal cortices of human AD brains [55]. It is now known that NF-κB preexists in the cytoplasm of cells in an inactive form bound to the inhibitor, IκB, as NF-κB:IκB complex [56]. Under certain circumstances, stimulation of cells by diverse inducers causes phosphorylation of cytosolic NF-κB:IκB complex, then NF-κB is activated by phosphorylation of IκBα and released to the nucleus [57, 58]. In the nucleus, NF-κB dimers bind to target DNA elements and activate transcription of genes encoding proteins involved with innate immune or inflammation responses [59]. Thus, inhibition of the phosphorylation of IκBα and NF-κB p65 may be a valuable drug target to reduce stressful damage for AD patients. The most abundant form of the NF-κB family is the heterodimeric p65–p50 complex [57] and the best-characterized IκB proteins is IκBα [56]. Our result revealed that the levels of phosphorylation of NF-κB p65 and IκBα were significantly accentuated in the brain of AlCl_3_ + vehicle mice. IRN treatment decreased the phosphorylation of NF-κB p65 and IκBα, suggesting that IRN inhibited the activation of NF-κB signaling pathway induced by AlCl_3_ in mice.

## Conclusions

Our experimental results for the first time demonstrated that IRN alleviated learning and memory impairments induced by AlCl_3_ in mice. The protective effect of IRN against AlCl_3_-induced cognitive deficits is probably mediated, at least in part, through inhibiting the AChE activity and reducing the oxidative damage of brain tissue via inhibiting the activation of NF-κB signaling pathway. These results further confirmed the in vivo anti-AD effect of IRN and suggested that IRN exhibits multi-target therapeutic potential for the treatment of AD. It was concluded that IRN could protect the learning and memory function. Further investigations are needed to reveal the anti-AD effect mechanisms of IRN.

## Additional file

**Additional file 1.** Minimum standards of reporting checklist.

## Abbreviations

AChE: acetylcholinesterase
AD: Alzheimer’s disease
AlCl_3_: aluminum chloride
BuChE: butyrylcholinesterase
CAT: catalase
DTNB: 5,5′-dithiobis (2-nitrobenzoic acid)
CMC-Na: sodium carboxymethyl cellulose
GSH: glutathione
IRN: isorhynchophylline
MDA: malondialdehyde
MCI: mild cognitive impairment
NF-Κb: nuclear factor kappa B
p-IκBα: phospho-IκBα
p-p65: phospho-NF-κB p65
RAM: radial arm maze
ROS: reactive oxygen species
RMEs: reference memory errors
SDS-PAGE: sodium dodecyl sulfate polyacrylamide gel electrophoresis
SEM: standard error of the mean
SOD: superoxide dismutases
TBA: thiobarbituric acid
TNB: Sym-Trinitrobenzene
WMEs: working memory errors
